# Supplementary material for: The metaphase chromatin unit: A novel unit of higher-order chromosome organization in human mitotic cells
Source: PLoS One. 2026 Nov 13;21(11):e0361508. doi: 10.1371/journal.pone.0361508 (PMC13766260; doi:10.1371/journal.pone.0361508)
Supplement: Supplementary file 1 [file pone.0361508.s007.pdf]

ScreenIT Report

This is an automated report for 10.64898+2026.06.03.729997.pdf. This report was solicited by the PLOS One editorial team and provided by ScreenIT.

ScreenIT is an independent group of scientists developing automated tools that analyze academic papers. A set of automated tools screened your submitted manuscript and provided the report below. Each tool was created by your academic colleagues with the goal of helping authors.

The tools look for factors that are important for transparency, rigor and reproducibility, and we hope that the report might help you to improve reporting in your manuscript. Within the report you will find links to more information about the items that the tools check. These links include helpful papers, websites, or videos that explain why the item is important.

While our screening tools aim to improve and maintain quality standards they may, on occasion, miss nuances specific to your study type or flag something incorrectly. Each tool has limitations that are described on the ScreenIT website. The tools screen the main file for the paper; they are not able to screen supplements stored in separate files.

Please note that the Academic Editor had access to these comments while making a decision on your manuscript. The Academic Editor may ask that issues flagged in this report be addressed.

If you would like to provide feedback on the ScreenIT tool, please email the team at ScreenIt@bih-charite.de. If you have questions or concerns about the review process, please contact the PLOS One office at plosone@plos.org.

How to read the report:

- no action required

? - review for potential issues

! - a critical issue was found by a tool

n/a - may not be applicable to your paper

- more information about this item can be obtained using this link

Rigor

NIH rigor criteria are not applicable to paper type.
Generated by SciScore

Resources

Persistent unique identifiers for research resources, RRIDs, can be used to track these key resources though the literature. They are requested or required in many journals, but even if your journal does not require them, we strongly encourage the use of these for the readers of papers who wish to easily track down the key materials like antibodies, software tools, or mice.

Software and Algorithms	"Analyses used Python 3.10 with NumPy 1.26 and SciPy 1.13."	Python suggested: python-bx (RRID:SCR_024202. Please make sure this RRID suggestion is accurate before including in your manuscript.<br>NumPy suggested: NumPy (RRID:SCR_008633. Please make sure this RRID suggestion is accurate before including in your manuscript.<br>SciPy suggested: SciPy (RRID:SCR_008058. Please make sure this RRID suggestion is accurate before including in your manuscript.

Generated by SciScore.
SciScore only screens the text of the methods section; we apologize if the tool has missed items reported elsewhere in the paper or supplements.

Transparency

Open code	We found one or more references to open code in your manuscript that did not seem to be included in your code availability statement: "analysis code and the interactive mcu calculator are publicly available at https://github.com/goyal74/mcu-calculator and live at https://goyal74.github.io/mcu-calculator/.".If these are references to your own code, make sure to include them in the code availability statement. If these are code citations, make sure

		to clearly state that you used previously published code.
	Open data	"the calculator is permanently archived on zenodo (doi: 10.5281/zenodo.20532702; https://doi.org/10.5281/zenodo.20532702). source data: manuscript_chromosome_domain.xlsx supplementary table s1 (manuscript_chromosome_domain.xlsx"
	Self-acknowledged limitations	"Limitations. The five pooled cytogenetic studies span 1964 to 2013 with varying fixation protocols; the cross-study CV is 1.6 percent but inter-laboratory bias cannot be excluded. The arm-level analysis uses GRCh38 centromere positions; T2T-CHM13 acrocentric p-arms are smaller and reduce the arm-level correlation (Supplementary Section S2). Cross-species predictions (Supplementary Section S8) apply the human slope to non-human Mb values and are framed as falsifiability checks rather than independent slope tests. Direct future tests of the MCU would benefit from matched Hi-C and cytometric measurements in the same primary preparations."
	Conflict of interest statement	Thank you for stating any conflicts of interest.
	Funding statement	Thank you for reporting your funding source(s), or stating that there was no specific funding for this work.
n/a	Registration statement	A registered protocol makes the planned research project more transparent and can protect against bias. Please consider registering future confirmatory studies.

Generated by ODDPub, limitation-recognizer, rtransparent, trial-identifier.

Figures

	No bar graphs of continuous data	Thank you for not using bar graphs to present continuous data. Many different datasets and data distributions can lead to the same bar graph, and the actual data may suggest different conclusions from the summary statistics alone.
	No rainbow color maps	Thank you for not using rainbow colormaps. Rainbow color maps distort readers' perception of the data by introducing visual artifacts and are not accessible to colorblind readers.

Generated by JetFighter, Barzooka.
