# Supplementary material for: The metaphase chromatin unit: A novel unit of higher-order chromosome organization in human mitotic cells
Source: PLoS One. 2026 Nov 13;21(11):e0361508. doi: 10.1371/journal.pone.0361508 (PMC13766260; doi:10.1371/journal.pone.0361508)
Supplement: Supplementary file 4 [file pone.0361508.s009.docx]

**Response to Reviewers**

*Manuscript PONE-D-26-36374*

*"The Metaphase Chromatin Unit: A Novel Unit of Higher-Order Chromosome Organization in Human Mitotic Cells"*

Dear Editor and Reviewers,

We thank the editor and both reviewers for the constructive and encouraging evaluation of our manuscript. We have revised the manuscript to address the specific points raised by Reviewer 2 and the items flagged in the ScreenIT automated report. All changes are shown in the tracked-changes version of the manuscript; line numbers in this response refer to the clean revised manuscript. Our point-by-point responses follow.

**Reviewer 2**

**Reviewer comment:**

*"The manuscript by Goyal and Goyal is an interesting proposal to use a new Metaphase Chromatin unit which is useful for communication about chromosomes as well as comparing them. At the same time it should be taken with a bit grain of salt because it is not an absolute unit, i.e., the 1 MCU = 7.6 Mb DNA = 0.25 μm axial length is an average value that only applies to late metaphase. But it nicely connects DNA length to physical chromosome size and is also somewhat linked to the optical resolution. Also the clean relation L (μm) = 0.0329 x Mb + 0.043 obtained by combining different datasets and fitting their data, is useful and provides a relatively solid ground to relating DNA length and chromosome size. Finally, the actual impact of this idea is something that might develop over time in the field. I only have a few remarks about figure 6 that should be addressed: in this overview in the polymer biophysics part there are some statements that are not discussed/substantiated in the text. Specifically, the rigid rod with exponent 1 and the mitotic stretch tension are not discussed. Would either do this or drop the polymer biophysics part from the figure."*

**Response:**

We thank Reviewer 2 for the positive overall assessment and for identifying a genuine gap in our treatment of Figure 6. We agree that the polymer-biophysics panels of Figure 6 reference two concepts (the rigid-rod exponent and single-chromosome mitotic stretch tension) that were not substantiated in the main text. We have chosen to address this by adding explicit discussion of both items rather than removing the panels from the figure, since the polymer-biophysics viewpoint is central to the MCU's compatibility with the Daban multilayer framework that is otherwise extensively discussed in the revised manuscript.

Specifically, we have appended the following material to Section 4.5 (Relation to cross-species scaling laws):

"Figure 6 integrates polymer-biophysical scales alongside the cytogenetic, Hi-C, clinical, and evolutionary scales. Two of these panels merit explicit discussion. First, within the human genome, metaphase chromosome length scales approximately linearly with DNA content (empirical exponent 0.98 across 24 chromosomes), the rigid-rod limit of polymer scaling in which axial length is set by cross-sectional packing density rather than by fiber flexibility; this is distinct from the Kramer cross-species L proportional to Mb to the one-half exponent, which operates across the much larger range of genome sizes between species. Second, direct micromechanical measurements of single mitotic chromosomes have reported stretch tensions on the order of 100 to 1000 pN, with reversible extension in the lower range and plastic deformation above approximately 1 nN (Poirier et al. 2002; Marko 2008). These stretch tensions are consistent with the nucleosome-nucleosome contact energies estimated from the Daban multilayer model (Daban 2014; Daban 2024) and are difficult to reconcile with purely extended-fiber architectures, providing an independent physical argument in favour of the dense-packing framework underlying the MCU."

Two new references supporting this addition have been added:

• Poirier MG, Eroglu S, Marko JF (2002). The bending rigidity of mitotic chromosomes. Molecular Biology of the Cell 13(6):2170-2179. PMID: 12058078.

• Marko JF (2008). Micromechanical studies of mitotic chromosomes. Chromosome Research 16(3):469-497. PMID: 18461485.

We also appreciate Reviewer 2's observation that the MCU is an average value appropriate to the late-prometaphase / early-metaphase window of standard clinical cytogenetic preparations rather than an absolute structural quantum. The revised manuscript now makes this scope explicit in Section 3.7 and the Conclusions, framing the MCU as an operational chromosome-scale scaling reference rather than a new level of the chromatin hierarchy, and acknowledging the biological variability of metaphase dimensions in a dedicated caveat paragraph.

**ScreenIT Report (automated screening)**

**1. Open code — explicit code availability statement**

***ScreenIT comment:***

*"We found one or more references to open code in your manuscript that did not seem to be included in your code availability statement: analysis code and the interactive MCU calculator are publicly available at https://github.com/goyal74/mcu-calculator ... If these are references to your own code, make sure to include them in the code availability statement."*

**Response:**

A new "Code Availability Statement" subsection has been added to the end-matter immediately following the Data Availability Statement. It explicitly names the GitHub repository (https://github.com/goyal74/mcu-calculator), the live calculator deployment (https://goyal74.github.io/mcu-calculator/), the Zenodo permanent archive (DOI: 10.5281/zenodo.20532702), the MIT License, and the fact that no previously published code was reused. All software dependencies are listed with their RRIDs.

**2. Software RRIDs — Python, NumPy, SciPy**

***ScreenIT comment:***

*"Python suggested python-bx (RRID:SCR_024202) ... NumPy suggested (RRID:SCR_008633) ... SciPy suggested (RRID:SCR_008058). Please make sure this RRID suggestion is accurate before including in your manuscript."*

**Response:**

We have verified the RRIDs against the SciCrunch Resource Identification Portal and added them parenthetically in Section 2.4 (Statistical analysis). The sentence now reads: "Analyses used Python 3.10 (RRID:SCR_024202) with NumPy 1.26 (RRID:SCR_008633) and SciPy 1.13 (RRID:SCR_008058)." The RRIDs are also listed in the new Code Availability Statement.

**3. Other ScreenIT items**

The remaining ScreenIT items received positive assessments and required no action: open data (Zenodo archive and Supplementary Table S1 Excel file acknowledged); self-acknowledged limitations (dedicated Limitations subsection acknowledged); conflict-of-interest statement (present); funding statement (present); no bar graphs on continuous data (confirmed); no rainbow colormaps (confirmed). Trial registration is not applicable as this study used only previously published, de-identified cytogenetic data and public reference assemblies.

**Summary of changes**

- Section 2.4 (Methods, Statistical analysis): added RRIDs for Python, NumPy, SciPy.
- Section 4.5 (Discussion, Cross-species scaling laws): added one paragraph discussing the rigid-rod exponent and the single-chromosome mitotic stretch tension referenced in Figure 6, with two new citations (Poirier et al. 2002; Marko 2008).
- End-matter: added a new Code Availability Statement subsection immediately after the Data Availability Statement, explicitly naming the GitHub repository, the live calculator URL, the Zenodo DOI, the license, and the software RRIDs.
- No changes were made to figures, tables, the regression results, the MCU definition, the clinical microdeletion classification, the interactive calculator, or the public data repositories.

We thank the editor and both reviewers again for the care taken with our manuscript and for the suggestions that have improved it. We hope that the revisions address all concerns raised and that the manuscript is now suitable for publication in PLOS ONE.

Sincerely,

**Ravi Goyal, MD, PhD, MBA, FAHA**

Vice Chair of Research and Professor of Obstetrics and Gynecology

University of Arizona College of Medicine — Tucson

Email: goyalr@arizona.edu | ORCID: 0000-0003-2940-0051
