# Supplementary material for: The metaphase chromatin unit: A novel unit of higher-order chromosome organization in human mitotic cells
Source: PLoS One. 2026 Nov 13;21(11):e0361508. doi: 10.1371/journal.pone.0361508 (PMC13766260; doi:10.1371/journal.pone.0361508)
Supplement: Peer review history

# Peer review: Decision Letter 0

**DOI:** 10.1371/journal.pone.0361508.r001

**Authors:** Claude Prigent

29 Sep 2026

Dear Dr. Goyal,

Specifically

- Please provide answers regarding the IA comment regarding software and algorithms, particular “Analyses used Python 3.10 with NumPy 1.26 and SciPy 1.13.”

- Also, please answer the second comment “references to open code in your manuscript did not seem to be included in your code availability statement”.

- Eventually answer reviewer 2 comments regarding figure 6.

Please submit your revised manuscript by Oct 29 2026 11:59PM. If you will need more time than this to complete your revisions, please reply to this message or contact the journal office at plosone@plos.org. When you're ready to submit your revision, log on to https://www.editorialmanager.com/pone/ and select the 'Submissions Needing Revision' folder to locate your manuscript file.

- A letter that responds to each point raised by the academic editor and reviewer(s). You should upload this letter as a separate file labeled 'Response to Reviewers'.
- A marked-up copy of your manuscript that highlights changes made to the original version. You should upload this as a separate file labeled 'Revised Manuscript with Track Changes'.
- An unmarked version of your revised paper without tracked changes. You should upload this as a separate file labeled 'Manuscript'.

If you would like to make changes to your financial disclosure, please include your updated statement in your cover letter. Guidelines for resubmitting your figure files are available below the reviewer comments at the end of this letter.

If applicable, we recommend that you deposit your laboratory protocols in protocols.io to enhance the reproducibility of your results. Protocols.io assigns your protocol its own identifier (DOI) so that it can be cited independently in the future. For instructions see: https://journals.plos.org/plosone/s/submission-guidelines#loc-laboratory-protocols. Additionally, PLOS One offers an option for publishing peer-reviewed Lab Protocol articles, which describe protocols hosted on protocols.io. Read more information on sharing protocols at https://plos.org/protocols?utm_medium=editorial-email&utm_source=authorletters&utm_campaign=protocols.

As the corresponding author, your ORCID iD is verified in the submission system and will appear in the published article. PLOS supports the use of ORCID, and we encourage all coauthors to register for an ORCID iD and use it as well. Please encourage your coauthors to verify their ORCID iD within the submission system before final acceptance, as unverified ORCID iDs will not appear in the published article. *Only*  the individual author can complete the verification step; PLOS staff *cannot*  verify ORCID iDs on behalf of authors.

We look forward to receiving your revised manuscript.

Kind regards,

Claude Prigent

Academic Editor

PLOS One

Journal Requirements:

When submitting your revision, we need you to address these additional requirements.

1. Please ensure that your manuscript meets PLOS ONE's style requirements, including those for file naming. The PLOS ONE style templates can be found at

https://journals.plos.org/plosone/s/file?id=wjVg/PLOSOne_formatting_sample_main_body.pdf and

https://journals.plos.org/plosone/s/file?id=ba62/PLOSOne_formatting_sample_title_authors_affiliations.pdf.

2 We note that your Data Availability Statement is currently as follows: [All data is submitted as supplementary material. ]

Please confirm at this time whether or not your submission contains all raw data required to replicate the results of your study. Authors must share the “minimal data set” for their submission. PLOS defines the minimal data set to consist of the data required to replicate all study findings reported in the article, as well as related metadata and methods (https://journals.plos.org/plosone/s/data-availability#loc-minimal-data-set-definition).

For example, authors should submit the following data:

- The values behind the means, standard deviations and other measures reported;

- The values used to build graphs;

- The points extracted from images for analysis.

Authors do not need to submit their entire data set if only a portion of the data was used in the reported study.

If your submission does not contain these data, please either upload them as Supporting Information files or deposit them to a stable, public repository and provide us with the relevant URLs, DOIs, or accession numbers. For a list of recommended repositories, please see https://journals.plos.org/plosone/s/recommended-repositories.

If there are ethical or legal restrictions on sharing a de-identified data set, please explain them in detail (e.g., data contain potentially sensitive information, data are owned by a third-party organization, etc.) and who has imposed them (e.g., an ethics committee). Please also provide contact information for a data access committee, ethics committee, or other institutional body to which data requests may be sent. If data are owned by a third party, please indicate how others may request data access.

3. If the reviewer comments include a recommendation to cite specific previously published works, please review and evaluate these publications to determine whether they are relevant and should be cited. There is no requirement to cite these works unless the editor has indicated otherwise.

4. Please review your reference list to ensure that it is complete and correct. If you have cited papers that have been retracted, please include the rationale for doing so in the manuscript text, or remove these references and replace them with relevant current references. Any changes to the reference list should be mentioned in the rebuttal letter that accompanies your revised manuscript. If you need to cite a retracted article, indicate the article’s retracted status in the References list and also include a citation and full reference for the retraction notice.

[Note: HTML markup is below. Please do not edit.]

Reviewers' comments:

Reviewer's Responses to Questions

**Comments to the Author**

1. Is the manuscript technically sound, and do the data support the conclusions?

Reviewer #1: Yes

Reviewer #2: Yes

**********

2. Has the statistical analysis been performed appropriately and rigorously?

Reviewer #1: Yes

Reviewer #2: Yes

**********

3. Have the authors made all data underlying the findings in their manuscript fully available?

The PLOS Data policy

Reviewer #1: Yes

Reviewer #2: Yes

**********

4. Is the manuscript presented in an intelligible fashion and written in standard English?

Reviewer #1: Yes

Reviewer #2: Yes

**********

Reviewer #1: This is an automated report for PONE-D-26-36374. This report was solicited by the PLOS One editorial team and provided by ScreenIT. ScreenIT is an independent group of scientists developing automated tools that analyze academic papers. A set of automated tools screened your submitted manuscript and provided the report below. Each tool was created by your academic colleagues with the goal of helping authors. The tools look for factors that are important for transparency, rigor and reproducibility, and we hope that the report might help you to improve reporting in your manuscript. Within the report you will find links to more information about the items that the tools check. These links include helpful papers, websites, or videos that explain why the item is important. While our screening tools aim to improve and maintain quality standards they may, on occasion, miss nuances specific to your study type or flag something incorrectly. Each tool has limitations that are described on the ScreenIT website. The tools screen the main file for the paper; they are not able to screen supplements stored in separate files. Please note that the Academic Editor had access to these comments while making a decision on your manuscript. The Academic Editor may ask that issues flagged in this report be addressed. If you would like to provide feedback on the ScreenIT tool, please email the team at ScreenIt@bih-charite.de. If you have questions or concerns about the review process, please contact the PLOS One office at plosone@plos.org.

Reviewer #2: The manuscript by Goyal and Goyal is an interesting proposal to use a new Metaphase Chromatin unit which is useful for communication about chromosomes as well as comparing them. At the same time it should be taken with a bit grain of salt because it not an absolute unit i.e. the 1 MCU = 7.6 Mb DNA = 0.25 μm axial length is an average value that only applies late metaphase. But it nicely connects DNA length to fysical chromosomes size and is also somewhat linked to the optical resolution. Also the clean relation: L (μm) = 0.0329 x Mb + 0.043 obtained by combining different datasets and fitting their data, is useful and provide a relatively solid ground to relating DNA length and chromosome size. Finally, the actual impact of this idea is something that might develop over time in the field.

I only have a few remarks about figure 6 that should be addressed: in this overview in the polymer biophysics part there are some statements that are not discussed/substaniated in the text. Specifically, the rigid rod with exponent 1 and the mitotic stretch tension are not discussed. Would either do this or drop the polymer biophysics part from the figure.

**********

what does this mean?). If published, this will include your full peer review and any attached files.

If you choose “no”, your identity will remain anonymous but your review may still be made public.

**Do you want your identity to be public for this peer review?**  For information about this choice, including consent withdrawal, please see our Privacy Policy

Reviewer #1: No

Reviewer #2: No

**********

[NOTE: If reviewer comments were submitted as an attachment file, they will be attached to this email and accessible via the submission site. Please log into your account, locate the manuscript record, and check for the action link "View Attachments". If this link does not appear, there are no attachment files.]

To ensure your figures meet our technical requirements, please review our figure guidelines: https://journals.plos.org/plosone/s/figures

You may also use PLOS’s free figure tool, NAAS, to help you prepare publication quality figures: https://journals.plos.org/plosone/s/figures#loc-tools-for-figure-preparation.

NAAS will assess whether your figures meet our technical requirements by comparing each figure against our figure specifications.

Attachment Submitted filename: report_10.64898+2026.06.03.729997.pdf

# Author comment: Author response to Decision Letter 1

**DOI:** 10.1371/journal.pone.0361508.r002

9 Oct 2026

Response to Reviewers

Manuscript PONE-D-26-36374

"The Metaphase Chromatin Unit: A Novel Unit of Higher-Order Chromosome Organization in Human Mitotic Cells"

Dear Editor and Reviewers,

We thank the editor and both reviewers for the constructive and encouraging evaluation of our manuscript. We have revised the manuscript to address the specific points raised by Reviewer 2 and the items flagged in the ScreenIT automated report. All changes are shown in the tracked-changes version of the manuscript; line numbers in this response refer to the clean revised manuscript. Our point-by-point responses follow.

Reviewer 2

Reviewer comment:

"The manuscript by Goyal and Goyal is an interesting proposal to use a new Metaphase Chromatin unit which is useful for communication about chromosomes as well as comparing them. At the same time it should be taken with a bit grain of salt because it is not an absolute unit, i.e., the 1 MCU = 7.6 Mb DNA = 0.25 μm axial length is an average value that only applies to late metaphase. But it nicely connects DNA length to physical chromosome size and is also somewhat linked to the optical resolution. Also the clean relation L (μm) = 0.0329 x Mb + 0.043 obtained by combining different datasets and fitting their data, is useful and provides a relatively solid ground to relating DNA length and chromosome size. Finally, the actual impact of this idea is something that might develop over time in the field. I only have a few remarks about figure 6 that should be addressed: in this overview in the polymer biophysics part there are some statements that are not discussed/substantiated in the text. Specifically, the rigid rod with exponent 1 and the mitotic stretch tension are not discussed. Would either do this or drop the polymer biophysics part from the figure."

Response:

We thank Reviewer 2 for the positive overall assessment and for identifying a genuine gap in our treatment of Figure 6. We agree that the polymer-biophysics panels of Figure 6 reference two concepts (the rigid-rod exponent and single-chromosome mitotic stretch tension) that were not substantiated in the main text. We have chosen to address this by adding explicit discussion of both items rather than removing the panels from the figure, since the polymer-biophysics viewpoint is central to the MCU's compatibility with the Daban multilayer framework that is otherwise extensively discussed in the revised manuscript.

Specifically, we have appended the following material to Section 4.5 (Relation to cross-species scaling laws):

"Figure 6 integrates polymer-biophysical scales alongside the cytogenetic, Hi-C, clinical, and evolutionary scales. Two of these panels merit explicit discussion. First, within the human genome, metaphase chromosome length scales approximately linearly with DNA content (empirical exponent 0.98 across 24 chromosomes), the rigid-rod limit of polymer scaling in which axial length is set by cross-sectional packing density rather than by fiber flexibility; this is distinct from the Kramer cross-species L proportional to Mb to the one-half exponent, which operates across the much larger range of genome sizes between species. Second, direct micromechanical measurements of single mitotic chromosomes have reported stretch tensions on the order of 100 to 1000 pN, with reversible extension in the lower range and plastic deformation above approximately 1 nN (Poirier et al. 2002; Marko 2008). These stretch tensions are consistent with the nucleosome-nucleosome contact energies estimated from the Daban multilayer model (Daban 2014; Daban 2024) and are difficult to reconcile with purely extended-fiber architectures, providing an independent physical argument in favour of the dense-packing framework underlying the MCU."

Two new references supporting this addition have been added:

• Poirier MG, Eroglu S, Marko JF (2002). The bending rigidity of mitotic chromosomes. Molecular Biology of the Cell 13(6):2170-2179. PMID: 12058078.

• Marko JF (2008). Micromechanical studies of mitotic chromosomes. Chromosome Research 16(3):469-497. PMID: 18461485.

We also appreciate Reviewer 2's observation that the MCU is an average value appropriate to the late-prometaphase / early-metaphase window of standard clinical cytogenetic preparations rather than an absolute structural quantum. The revised manuscript now makes this scope explicit in Section 3.7 and the Conclusions, framing the MCU as an operational chromosome-scale scaling reference rather than a new level of the chromatin hierarchy, and acknowledging the biological variability of metaphase dimensions in a dedicated caveat paragraph.

ScreenIT Report (automated screening)

1. Open code — explicit code availability statement

ScreenIT comment:

"We found one or more references to open code in your manuscript that did not seem to be included in your code availability statement: analysis code and the interactive MCU calculator are publicly available at https://github.com/goyal74/mcu-calculator ... If these are references to your own code, make sure to include them in the code availability statement."

Response:

A new "Code Availability Statement" subsection has been added to the end-matter immediately following the Data Availability Statement. It explicitly names the GitHub repository (https://github.com/goyal74/mcu-calculator), the live calculator deployment (https://goyal74.github.io/mcu-calculator/), the Zenodo permanent archive (DOI: 10.5281/zenodo.20532702), the MIT License, and the fact that no previously published code was reused. All software dependencies are listed with their RRIDs.

2. Software RRIDs — Python, NumPy, SciPy

ScreenIT comment:

"Python suggested python-bx (RRID:SCR_024202) ... NumPy suggested (RRID:SCR_008633) ... SciPy suggested (RRID:SCR_008058). Please make sure this RRID suggestion is accurate before including in your manuscript."

Response:

We have verified the RRIDs against the SciCrunch Resource Identification Portal and added them parenthetically in Section 2.4 (Statistical analysis). The sentence now reads: "Analyses used Python 3.10 (RRID:SCR_024202) with NumPy 1.26 (RRID:SCR_008633) and SciPy 1.13 (RRID:SCR_008058)." The RRIDs are also listed in the new Code Availability Statement.

3. Other ScreenIT items

The remaining ScreenIT items received positive assessments and required no action: open data (Zenodo archive and Supplementary Table S1 Excel file acknowledged); self-acknowledged limitations (dedicated Limitations subsection acknowledged); conflict-of-interest statement (present); funding statement (present); no bar graphs on continuous data (confirmed); no rainbow colormaps (confirmed). Trial registration is not applicable as this study used only previously published, de-identified cytogenetic data and public reference assemblies.

Summary of changes

• Section 2.4 (Methods, Statistical analysis): added RRIDs for Python, NumPy, SciPy.

• Section 4.5 (Discussion, Cross-species scaling laws): added one paragraph discussing the rigid-rod exponent and the single-chromosome mitotic stretch tension referenced in Figure 6, with two new citations (Poirier et al. 2002; Marko 2008).

• End-matter: added a new Code Availability Statement subsection immediately after the Data Availability Statement, explicitly naming the GitHub repository, the live calculator URL, the Zenodo DOI, the license, and the software RRIDs.

• No changes were made to figures, tables, the regression results, the MCU definition, the clinical microdeletion classification, the interactive calculator, or the public data repositories.

We thank the editor and both reviewers again for the care taken with our manuscript and for the suggestions that have improved it. We hope that the revisions address all concerns raised and that the manuscript is now suitable for publication in PLOS ONE.

Sincerely,

Ravi Goyal, MD, PhD, MBA, FAHA

Vice Chair of Research and Professor of Obstetrics and Gynecology

University of Arizona College of Medicine — Tucson

Email: goyalr@arizona.edu | ORCID: 0000-0003-2940-0051

Attachment Submitted filename: Response_to_Reviewers_PLOS_ONE.docx

# Editor report: Decision Letter 1

**DOI:** 10.1371/journal.pone.0361508.r003

**Authors:** Claude Prigent

12 Oct 2026

The Metaphase Chromatin Unit: A Novel Unit of Higher-Order Chromosome Organization in Human Mitotic Cells

PONE-D-26-36374R1

Dear Dr. Goyal,

We’re pleased to inform you that your manuscript has been judged scientifically suitable for publication and will be formally accepted for publication once it meets all outstanding technical requirements.

Within one week, you’ll receive an e-mail detailing the required amendments. When these have been addressed, you’ll receive a formal acceptance letter and your manuscript will be scheduled for publication.

An invoice will be generated when your article is formally accepted. Please note, if your institution has a publishing partnership with PLOS and your article meets the relevant criteria, all or part of your publication costs will be covered. Please make sure your user information is up-to-date by logging into Editorial Manager at Editorial Manager® and clicking the ‘Update My Information' link at the top of the page. For questions related to billing, please contact billing support.

If your institution or institutions have a press office, please notify them about your upcoming paper to help maximize its impact. If they’ll be preparing press materials, please inform our press team as soon as possible -- no later than 48 hours after receiving the formal acceptance. Your manuscript will remain under strict press embargo until 2 pm Eastern Time on the date of publication. For more information, please contact onepress@plos.org.

Kind regards,

Claude Prigent

Academic Editor

PLOS One

Additional Editor Comments (optional):

Reviewers' comments:

# Editor report: Acceptance letter

**DOI:** 10.1371/journal.pone.0361508.r004

**Authors:** Claude Prigent

PONE-D-26-36374R1

PLOS One

Dear Dr. Goyal,

I'm pleased to inform you that your manuscript has been deemed suitable for publication in PLOS One. Congratulations! Your manuscript is now being handed over to our production team.

At this stage, our production department will prepare your paper for publication. This includes ensuring the following:

* All references, tables, and figures are properly cited

* All relevant supporting information is included in the manuscript submission,

* There are no issues that prevent the paper from being properly typeset

You will receive further instructions from the production team, including instructions on how to review your proof when it is ready. Please keep in mind that we are working through a large volume of accepted articles, so please give us a few days to review your paper and let you know the next and final steps.

Lastly, if your institution or institutions have a press office, please let them know about your upcoming paper now to help maximize its impact. If they'll be preparing press materials, please inform our press team within the next 48 hours. Your manuscript will remain under strict press embargo until 2 pm Eastern Time on the date of publication. For more information, please contact onepress@plos.org.

You will receive an invoice from PLOS for your publication fee after your manuscript has reached the completed accept phase. If you receive an email requesting payment before acceptance or for any other service, this may be a phishing scheme. Learn how to identify phishing emails and protect your accounts at https://explore.plos.org/phishing.

If we can help with anything else, please email us at customercare@plos.org.

Thank you for submitting your work to PLOS One and supporting open access.

Kind regards,

PLOS One Editorial Office Staff

on behalf of

Dr. Claude Prigent

Academic Editor

PLOS One
